# Enhanced Production of Active Photosynthetic and Biochemical Molecules in *Silybum marianum* L. Using Biotic and Abiotic Elicitors in Hydroponic Culture

**DOI:** 10.3390/molecules28041716

**Published:** 2023-02-10

**Authors:** Bismillah Mubeen, Ammarah Hasnain, Wang Jie, Hanxian Zheng, Willie J. G. M. Peijnenburg, Shahril Efzueni Rozali, Rabia Rasool, Syed Atif Hasan Naqvi, Muhammad Junaid Rao, Muhammad Amir Sohail, Mahmoud Moustafa, Mohammed Al-Shehri, Sally Negm

**Affiliations:** 1 Tobacco Research Institute of Chinese Academy of Agricultural Sciences, Qingdao 266101, China; 2 Institute of Molecular Biology and Biotechnology, The University of Lahore, Lahore 54590, Pakistan; 3 Institute of Environmental Sciences, Leiden University, 2333 CC Leiden, The Netherlands; 4 National Institute of Public Health and the Environment, Center for the Safety of Substances and Products, 3720 BA Bilthoven, The Netherlands; 5 School of Science and Psychology, Faculty of Arts and Science, International University of Malaya-Wales, City Campus, Kuala Lumpur 50480, Malaysia; 6 Department of Plant Pathology, Faculty of Agricultural Sciences and Technology, Bahauddin Zakariya University, Multan 60800, Pakistan; 7 State Key Laboratory for Conservation and Utilization of Subtropical Agro-Bioresources, Guangxi Key Laboratory of Sugarcane Biology, College of Agriculture, Guangxi University, Nanning 530006, China; 8 Hubei Key Laboratory of Plant Pathology, Huazhong Agricultural University, Wuhan 430070, China; 9 Department of Biology, Faculty of Science, King Khalid University, Abha 62529, Saudi Arabia; 10 Department of Botany and Microbiology, Faculty of Science, South Valley University, Qena 83523, Egypt; 11 Department of Life Sciences, College of Science and Art MahyelAseer, King Khalid University, Abha 62529, Saudi Arabia; 12 Unit of Food Bacteriology, Central Laboratory of Food Hygiene, Ministry of Health, Branch in Zagazig, Sharkia 11118, Egypt

**Keywords:** molecules, bioactivity, natural products, elicitation, methyl jasmonate, bio-active natural compounds

## Abstract

Elicitors are stressors that activate secondary pathways that lead to the increased production of bioactive molecules in plants. Different elicitors including the fungus *Aspergillus niger* (0.2 g/L), methyl jasmonate (MeJA, 100 µM/L), and silver nanoparticles (1 µg/L) were added, individually and in combination, in a hydroponic medium. The application of these elicitors in hydroponic culture significantly increased the concentration of photosynthetic pigments and total phenolic contents. The treatment with MeJA (methyl jasmonate) (100 µM/L) and the co-treatment of MeJA and AgNPs (silver nanoparticles) (100 µM/L + 1 µg/L) exhibited the highest chlorophyll a (29 µg g^−1^ FW) and chlorophyll b (33.6 µg g^−1^ FW) contents, respectively. The elicitor MeJA (100 µM/L) gave a substantial rise in chlorophyll a and b and total chlorophyll contents. Likewise, a significant rise in carotenoid contents (9 µg/g FW) was also observed when subjected to meJA (100 µM/L). For the phenolic content, the treatment with meJA (100 µM/L) proved to be very effective. Nevertheless, the highest production (431 µg/g FW) was observed when treated with AgNPs (1 µg/L). The treatments with various elicitors in this study had a significant effect on flavonoid and lignin content. The highest concentration of flavonoids and lignin was observed when MeJA (100 mM) was used as an elicitor, following a 72-h treatment period. Hence, for different plant metabolites, the treatment with meJA (100 µM/L) and a co-treatment of MeJA and AgNPs (100 µM/L + 1 µg/L) under prolonged exposure times of 120–144 h proved to be the most promising in the accretion of valuable bioactive molecules. The study opens new insights into the use of these elicitors, individually or in combination, by using different concentrations and compositions.

## 1. Introduction

Herbs are plants or plant parts used to treat many physiological diseases as they possess various phytochemical and remedial properties [1]. In different countries, local physicians used different herbal remedies as therapeutic agents. The herbal plant *Silybum marianum* L. has been found very effective to cure liver disorders. *S. marianum* L. (Milk thistle) is a herbaceous broadleaf plant that grows both annually and biennially and is a member of the *Asteraceae* family [2]. *S. marianum* has recently gained much attention from the scientific community as it possesses medicinal and therapeutic value [3]. The significance of *S. marianum* and subsequently its active constituents are evident from the list of diseases for which the plant extracts have been used as therapeutic agents [4]. The diseases such as cancer, anorexia, diabetes, hepatitis, hemorrhoids, cirrhosis, malaria, spleen disease, and estrogen-related diseases are found to be treated with the pharmacologically important metabolites obtained from *S. marianum* [2,3,4,5]. This plant is well-known for curing biliary and liver conditions, including liver cancer [6].

Chlorophyll pigments (chlorophyll a and chlorophyll b) are photosynthetic pigments that give plants their green color. Chlorophyll has antioxidant properties in human nutrition, and its bright color makes food more appealing to eat [7,8]. It has also been found to have anti-cancer qualities because it can prevent some carcinogenic substances from being absorbed into the gastrointestinal system [9].

Photosystems in plants are protected by carotenoids, which allow them to engage in photosynthesis [10]. Carotenoids are potent antioxidants with a wide range of health benefits, including pro-vitamin A activity, cancer prevention, improved cognitive function, and improved eye and cardiovascular health [11,12]. Immunomodulation and the prevention of degenerative diseases have also been connected to carotenoids [13]. Carotenoid-rich diets have also been linked to a lower incidence of various diseases in epidemiological studies [14].

Phenolics are a wide category of phytochemicals that includes, among other things, phenolic acids and flavonoids [15]. They are powerful antioxidants with anti-inflammatory, anti-obesity, and anti-cancer activities [12,13,14,15,16]. They have also been demonstrated to help prevent diabetes and cardiovascular disease [16]. It is the main bioactive ingredient in *S. marianum* to cure numerous deadly illnesses because it includes flavonoids and flavonolignans, which are recognized to have therapeutic potential in *S. marianum* [17,18]. Photosynthetic pigments play a fundamental role in plant defense response by increasing phenolic contents in plants [19]. In plant cells, phenolic compounds play a variety of roles, including defense against insect attack and infection by disease agents such as fungi, bacteria, and viruses [20,21], DNA protection from oxidative damage, and reductions in photo-oxidative damage to photosystems [22,23].

Lignin and flavonoids have antioxidant and antimicrobial properties and have potential use in the production of pharmaceuticals and biomedical applications. Its biocompatibility, ability to absorb UV light, antioxidant and antimicrobial activities, and ability to improve the mechanical strength of processed biomaterials make it a promising candidate for pharmaceutical and biomedical applications. Research suggests that lignin and flavonoids could be used in the development of biomedical hydrogels, drug delivery vehicles, or biocomposites for tissue engineering and wound healing [24,25,26].

Genetic, physiological, ecological, and environmental variables all have an impact on the *S. marianum* secondary compounds’ production that was grown in vivo [27,28]. All of these factors cause variations in the quality and quantity of *S. marianum* medicinal formulations that are commercially available. As a result, a strategy for growing *S. marianum* in vitro with standardized quantities of physiologically active substances is needed to optimize its production [29,30]. The use of culture media has been the most popular strategy employed in recent decades to obtain improved yields in the production system [31]. Recent research suggests that several bioactive molecules and plant secondary metabolites along with various reactive oxygen species are involved in signal transduction at the cellular level when a host plant is faced with any type of biotic or abiotic stress [30].

Different elicitor treatments can be used to induce the formation of secondary metabolites in plant cells in vitro [32]. In response to elicitors, plants produce bioactive chemicals as a natural defensive mechanism. Elicitors are stressors that activate secondary pathways that lead to the production of bioactive substances. Elicitors are classified as either abiotic or biotic based on their natural state. Physical agents (e.g., UV irradiation, temperature, mechanical injury) and chemical compounds (e.g., salicylic acid (SA), jasmonic acid (JA), and methyl jasmonate) are examples of non-biological elicitors (MeJA) [33,34,35,36]. Biotic elicitors come from a microbial or plant source, such as yeast extract, and have a biological origin [37]. In vitro, abiotic and biotic elicitors were used to rapidly produce a large number of bioactive chemicals [33]. Elicitation can also affect the creation of other bioactive substances such as vitamins and plant pigments (chlorophylls and carotenoids), affecting plant organoleptic quality, particularly color [14].

Nanotechnology is an emerging technology, and the use of nanoparticles (NPs) as elicitors shows promising effects to increase secondary products in the body of plants [38]. The authors of reference [39] observed nine different types of mono- and bimetallic alloy metal NPs including gold (Au), copper (Cu), silver (Ag), silver–copper (1:3), silver–copper (3:1), silver–copper (1:3), silver–copper (3:1), silver–gold (1:3), and silver–gold (3:1) which exerted their influence on the stem and root growth, seed growth, and biochemical levels of the plant *S. marianum*. When the seeds were given NP suspension treatment, their growth was noticeably enhanced especially with silver nanoparticle (AgNP) suspension. The effect of AgNPs was found to be more significant among all the NPs used in mapping the conclusions of different mono- and bimetallic NPs on medicinal plant species. NPs have a significant impact on the development of hairy roots and seeds of many plants such as *Lactuca sativa*, *Brassica napus*, *Zea mays*, *Raphanus sativus*, *Cucumis sativus*, and *Lolium multiflorum*. The authors of reference [40] observed that AgNPs have a favorable impact on *S. marianum* as the concentration of secondary metabolites was remarkably increased in the medium. In another study, AgNPs used for *Brassica juncea* seeds increased seedling vigor, root, shoot height, and weight [41]. Hence, AgNPs are crucial for plant growth because they regulate numerous metabolic tracks which significantly affect phyto- chemical levels in plant cells.

*Aspergillus niger* is also known to play an important role as a biotic fungal elicitor for the enhancement of the synthesis of secondary metabolites in laboratory cultures [39,40]. In addition to this, methyl jasmonate (MeJA), a plant growth regulator, is also regarded as a useful biotic elicitor against different stresses. When there is any damage in plant cells, jasmonic acid and MeJA are produced in the damaged part of the plant [42].

Using a soilless growing method called hydroponics, highly nutritious media are used to cultivate plants with enough dissolved oxygen [43,44]. It is a method of soilless cultivation where the roots are submerged in water H_2_O enriched with mineral elements while being fixed by growing materials such as pebbles, clay, or perlite [45]. Precise and regular surveillance is necessary to improve the formation of liquid fertilizer to enhance plant production [46]. A hydroponic system can be employed to augment the production of medicinally important compounds in plants by using different elicitors. Elicitation is a promising technique for increasing phytochemical contents by activating the defense mechanism of plants [34].

There is however little information on the effects of elicitors on these metabolites in medicinal plants, specifically *S. marianum* (L.). This study aimed to investigate the response of *S. marianum* (L.) to elicitors in a hydroponics culture. Considering the medicinal importance of *S. marianum*’s bioactive compounds and due to the lack of information on the effects of elicitors’ application on chlorophyll, carotenoid, and total phenolic contents in *S. marianum*, the current study was designed to investigate biotic and abiotic elicitors’ effects on the accretion of these bioactive compounds. Moreover, a combination of different elicitors has not been studied previously on hydroponically grown medicinal plants. Therefore, in the current study, three different elicitors, i.e., MeJA, fungal elicitors, and AgNPs, were utilized to examine their impact on the buildup of bioactive chemicals in hydroponic environments, both singly and even in mixture form.

## 2. Results

The applied treatments had a significant impact on the chlorophyll a, b, ab, total chlorophyll, carotenoids, lignins, and flavonoid contents in *S. marianum* under hydroponic culture.

### 2.1. Weight of Treated Plants

For different treatments, no significant change in plant weight was observed, and a pairwise comparison was performed which showed no significance among the treatments used under hydroponics.

### 2.2. Chlorophyll a, b, ab and Total Chlorophyll Contents in Treated Plants

**Figure 1:**
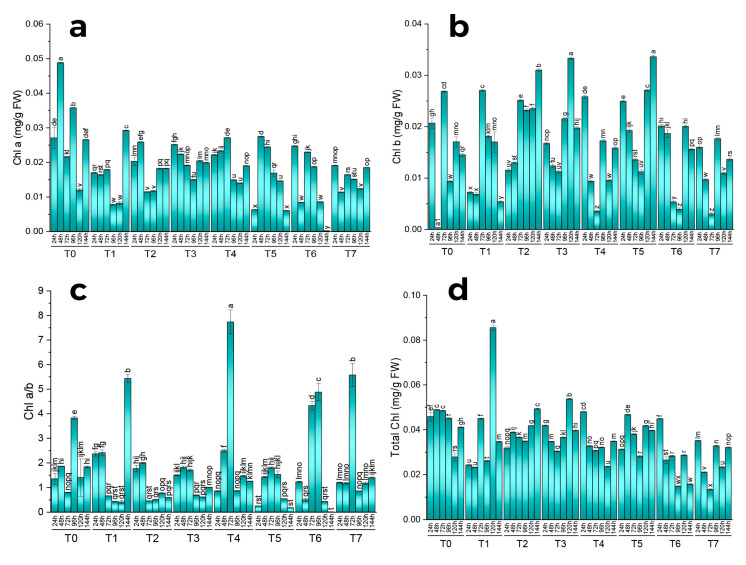
Effect of different treatments of elicitors on chlorophyll a (**a**), b (**b**), and ab (**c**) and total chlorophyll (**d**) contents of *Silybum marianum* in hydroponics where harvest time on x-axis, first = one day (24 h), second = 48 h, third = 72 h, fourth = 96 h, fifth = 120 h, sixth = 144 h, where (T0 = control, T1 = methyl jasmonate, T2 = silver nanoparticles, T3 = fungal elicitors, T4 = methyl jasmonate + fungal elicitors, T5 = methyl jasmonate + silver nanoparticles, T6 = fungal elicitors + silver nanoparticles, T7 = methyl jasmonate + silver nanoparticles + fungal elicitors); different letters on the bars indicate a difference that was significant at *p* > 0.05; Tukey test.

**Figure 2:**
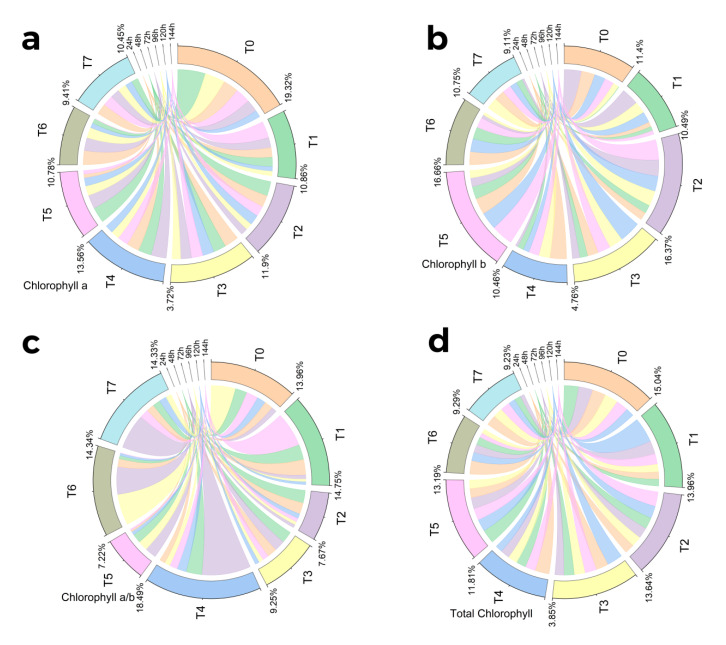
Chord diagram showing effect of different treatments of elicitors on chlorophyll a (**a**), b (**b**), and ab (**c**) and total chlorophyll (**d**) contents of *Silybum marianum* in hydroponics where harvest time on x-axis, first = one day (24 h), second = 48 h, third = 72 h, fourth = 96 h, fifth = 120 h, sixth = 144 h, where (T0 = control, T1 = methyl jasmonate, T2 = silver nanoparticles, T3 = fungal elicitors, T4 = methyl jasmonate + fungal elicitors, T5 = methyl jasmonate + silver nanoparticles, T6 = fungal elicitors + silver nanoparticles, T7 = methyl jasmonate + silver nanoparticles + fungal elicitors).

All the treatments showed a significant effect on chlorophyll content. The highest chlorophyll content was found in control plants after 48 h. However, as the treatment time increased, the highest chlorophyll content was found in plants treated with methyl jasmonate (100 µM/L) after 144 h. Both primacy and insignificance among the replications were revealed by the pairwise comparison of the harvests and therapies (Figure 1a and Figure 2a).

A considerable impact of various treatments was found on chlorophyll b content. The commodity with the highest concentration of chlorophyll b was AgNP (1 ppm/L)-treated plants after 120 h, whereas, after 144 h, the co-treatment of MeJA and AgNPs (100 µM/L + 1 ppm/L) was found to be more effective in enhancing chlorophyll b content. The fungal elicitors (0.2 g/L) also caused an increase in chlorophyll b content after 72 h and 120 h of treatment. MeJA (100 µM/L) also caused an increase in chlorophyll b content after 72 h of treatment. Both primacy and insignificance among the replications were revealed by the pairwise comparison of the harvests and therapies (Figure 1b and Figure 2b).

All the treatments showed a significant effect on chlorophyll a and b content. The highest concentration of chlorophyll ab was noted under the combined treatment of MeJA (100 µM/L) and fungal elicitors after 72 h. However, after long-term exposure of 144 h, MeJA (100 µM/L) application was more effective in enhancing chlorophyll ab. The combined treatment of fungal elicitors (0.2 g/L) and AgNPs (1 ppm) and MeJA (100 µM/L) also increased chlorophyll ab content after 72 h of treatment. Moreover, the combined effect of fungal elicitors (0.2 g/L) and AgNPs (1 ppm) also showed an increase in chlorophyll ab content after 72 h and 96 h of treatment. Both primacy and insignificance among the replications were revealed by the pairwise comparison of the harvests and therapies (Figure 1c and Figure 2c).

All the treatments showed a significant effect on the total chlorophyll content. Treatments were found effective only after 120 h, and among these, individual methyl jasmonate (100 µM/L) application showed the highest total chlorophyll content. Both primacy and insignificance among the replications were revealed by the pairwise comparison of the harvests and therapies (Figure 1d and Figure 2d).

### 2.3. Carotenoid, Total Phenolics, Lignins, and Flavonoids Content in Treated Plants

**Figure 3:**
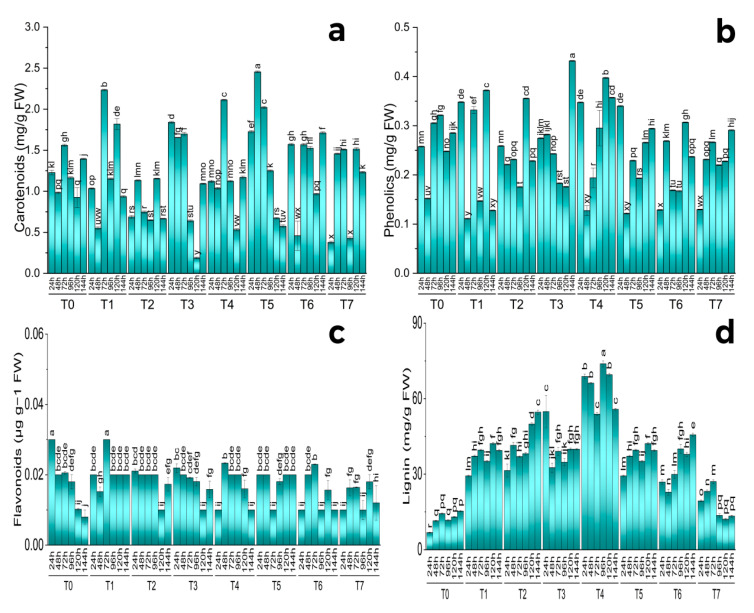
Effect of different treatments of elicitors on Carotenoids (**a**), Phenolics (**b**), Flavonoids (**c**), and Lignin (**d**) contents of *Silybum marianum* in hydroponics where harvest time on x-axis, first = one day (24 h), second = 48 h, third = 72 h, fourth = 96 h, fifth = 120 h, sixth = 144 h, where (T0 = control, T1 = methyl jasmonate, T2 = silver nanoparticles, T3 = fungal elicitors, T4 = methyl jasmonate + fungal elicitors, T5 = methyl jasmonate + silver nanoparticles, T6 = fungal elicitors + silver nanoparticles, T7 = methyl jasmonate + silver nanoparticles + fungal elicitors); different letters on the bars indicate a difference that was significant at *p* > 0.05; Tukey test.

**Figure 4:**
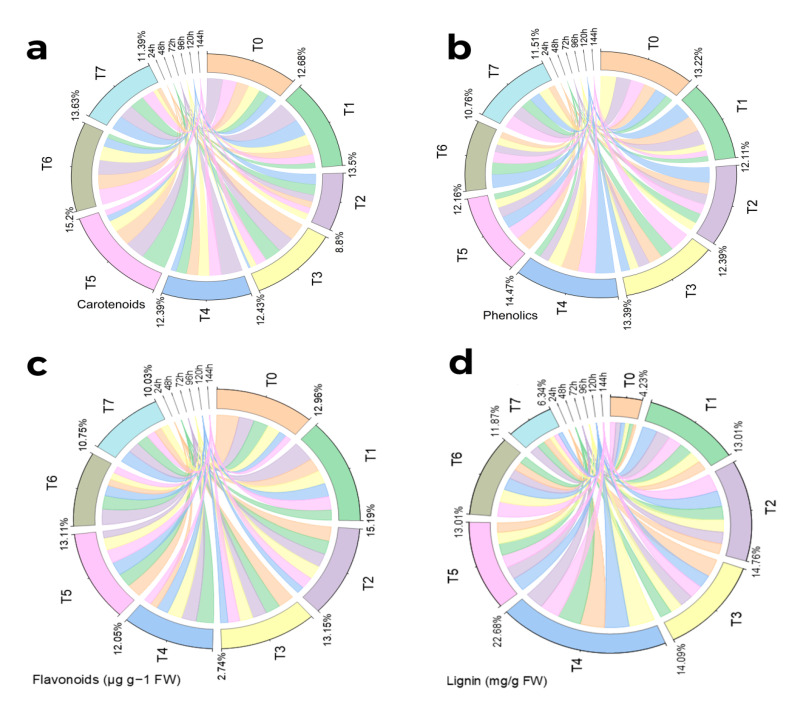
Chord diagram showing effect of different treatments of elicitors on *Carotenoids* (**a**), *Phenolics* (**b**), Flavonoids (**c**), and *Lignin* (**d**) contents of *Silybum marianum* in hydroponics where Harvest time on x-axis, First = One day (24 h), second = 48 h. Third = 72 h. Fourth = 96 h. Fifth = 120 h. Six = 144 h.where(T0 = control, T1 = methyl jasmonate, T2 = silver nanoparticles, T3 = fungal elicitors, T4 = methyl jasmonate + fungal elicitors, T5 = methyl jasmonate + silver nanoparticles, T6 = fungal elicitors + silver nanoparticles, T7 = methyl jasmonate + silver nanoparticles + fungal elicitors).

A substantial outcome of various treatments was found on carotenoid material. The highest carotenoid production has been observed under combined treatment of MeJA (100 µM/L) and silver nanoparticles (1 ppm) after 48 h of treatment. MeJA (100 µM/L) treatment also showed significantly increased production after 72 h and 120 h of treatment. Combined treatment of MeJA (100 µM/L) and fungal elicitors (0.2 g/L) also showed an increase in the carotenoid content after 72 h of treatment. AgNPs (1 ppm) showed a relentless increase in carotenoid content from 24 h to 72 h of treatment. An increase in carotenoid content was also observed under the combined treatment of fungal elicitors (0.2 g/L) and AgNPs (1 ppm) after 144 h. Both primacy and insignificance among the replications were revealed by the pairwise comparison of the harvests and therapies (Figure 3a and Figure 4a).

Under hydroponic conditions, various treatments showed a discernible increase in overall phenolics. Among different treatments, MeJA (100 µM/L), MeJA (100 µM/L) and fungal elicitors (0.2 g/L), and MeJA(100 µM/L) and AgNPs (1 ppm/L) implementations displayed an enhanced total phenolic concentration following 24 h. After 72 h, MeJA (100 µM/L) was more phenolic in total than other treatments. However, after 120 h, MeJA (100 µM/L) and MeJA (100 µM/L) and fungal elicitors (0.2 g/L) had been the most successful in increasing the total phenolic content. The highest total phenolic content was noticed after 144 h under AgNP treatment (1 ppm/L). Both primacy and insignificance among the replications were revealed by the pairwise comparison of the harvests and therapies. Pearson correlation for different phytochemical analyses of S. marianum in hydroponics culture with different treatments and principle component analysis of the effect of various treatments application in hydroponics culture to enhance phytochemicals of S. marianum has also been portrayed (Figure 3b and Figure 4b).

All the treatments showed a significant effect on the flavonoid content. The highest content was found in the plants after 72 h of treatment of MeJA (100 µM) and the control. All of the treatments almost showed good results in flavonoid production. The pairwise comparison of treatments and harvests showed both significance and non-significance among the treatments. Different letters on the bars show significant differences at *p* ≤ 0.05; Tukey test. The chord diagram is showing the average contribution of different treatments for the enhancement of flavonoid contents (Figure 3c and Figure 4c).

All the treatments showed a significant effect on the lignin content. The highest content was found in plants after 96 h under the combined treatment of MeJA and fungal elicitors (100 µM/L + 0.2 g/L). This treatment showed the best effect on lignin production in all six harvests. Fungal elicitors (0.2 g/L) and green-synthesized nanoparticles (1 ppm/L) separately also showed increased lignin content. Under fungal elicitor (0.2 g/L) treatment, the highest content was observed after 144 harvests. Green-synthesized nanoparticles (1 ppm/L) treatment showed the highest lignin content in harvest after 24 h. The combined treatment of MeJA and green-synthesized AgNPs (100 µM/L + 1 ppm/L) and fungal elicitors and green-synthesized AgNPs (0.2 g/L + 1 ppm/L) showed increased lignin content production in comparison to the control. The pairwise comparison of treatments and harvests showed both significance and non-significance among the treatments. Different letters on the bars show significant differences at *p* ≤ 0.05; Tukey test. The chord diagram shows the average contribution of different treatments for the enhancement of lignin content (Figure 3d and Figure 4d).

### 2.4. Principal Component Analysis

**Figure 5:**
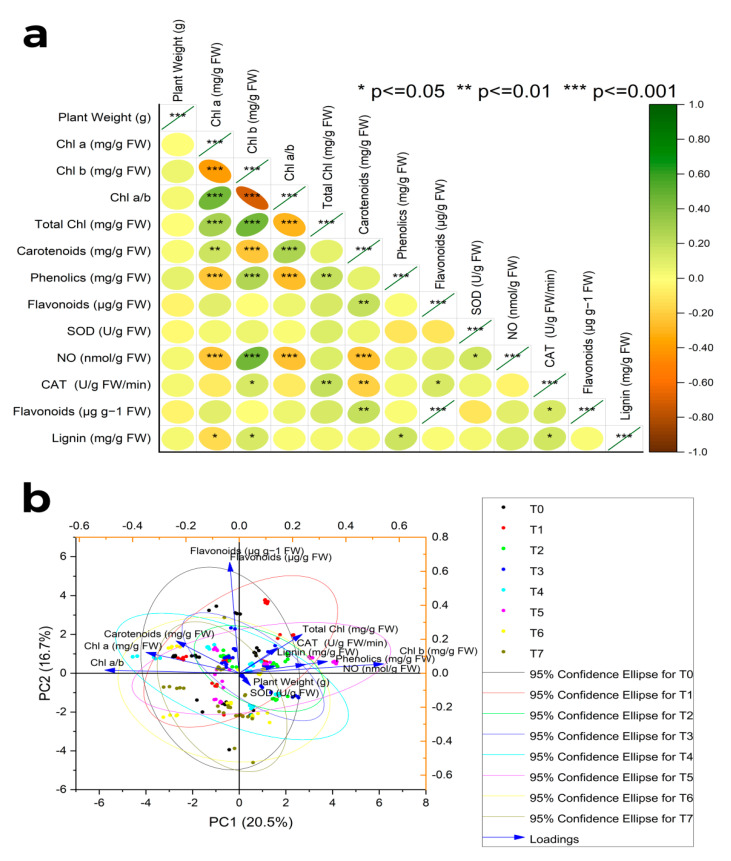
(**a**) Pearson correlation for different phytochemical analysesof *Silybum marianum* in hydroponics culture with different treatments where (T0 = control, T1 = methyl jasmonate, T2 = silver nanoparticles, T3 = fungal elicitors, T4 = methyl jasmonate + fungal elicitors, T5 = methyl jasmonate + silver nanoparticles, T6 = fungal elicitors + silver nanoparticles, T7 = methyl jasmonate + silver nanoparticles + fungal elicitors). (**b**) Principal component analysis of the effect of various treatments application in hydroponics culture to enhance phytochemicals of *Silybum marianum*.

There is a high correlation among the various treatments, viz., (T0 = control, T1 = methyl jasmonate, T2 = silver nanoparticles, T3 = fungal elicitors, T4 = methyl jasmonate + fungal elicitors, T5 = methyl jasmonate + silver nanoparticles, T6 = fungal elicitors + silver nanoparticles, T7 = methyl jasmonate + silver nanoparticles + fungal elicitors) and the enhanced production of various secondary molecules in *S. marianum*. The first principal component (PC1) revealed 20.5% of the total variation. The PC2 explained 16.7% of the total variation in the enhanced production of various molecules by different treatments. The loading plots demonstrate that the relationships among various treatments with the enhanced production of various secondary molecules at different locations with a <90° angle of vectors are positively correlated and with a >90°angle of vectors are not correlated (Figure 5a,b).

## 3. Discussion

It has been a long-standing practice to treat patients using medicinal plants to treat a variety of ailments. Plant small molecules such as photosynthetic pigments and carotenoids play a key role in photosynthesis. The elicitation technique has commonly been used to increase the production of plants’ important bioactive compounds in parts of plant cells with special reference to medicinal plants [47]. In the present study, different types of elicitors (abiotic and biotic) have been used to enhance the production of these valuable compounds by *S. marianum* [48,49]. The most commonly used and effective elicitors for the stimulation of secondary metabolite production are methyl jasmonate (MeJA), fungal cells, and metallic nanoparticles [50,51]. Moreover, among different culture systems, scientists and growers claim that hydroponic systems allow plants to increase their capabilities for continuous production in a short growing period, require less space, and allow plants to be grown anywhere with a regulated growth environment [52,53]. The hydroponic technique is not seasonal, and just a few investigations on *S. marianum* in hydroponics have been conducted [54]. The use of elicitors under such controlled conditions is very useful for the enhanced production of secondary metabolites. MeJA and fungal elicitors increased bioactive compound production in in vitro cultures of *Stemona* sp. due to the increased production of stamina alkaloids [55]. Our findings are in line with the authors of references [21,56], where MeJA significantly increased the production of secondary metabolites such as total phenolics and flavonoid contents [34,57].

MeJA is considered a signaling molecule that stimulates the accumulation of essential phyto-compounds including chlorophyll and carotenoids [37]. Our results show enhanced photosynthetic pigments under the influence of MeJA, which coincides with the authors of reference [58] who reported a similar study of increased photosynthetic activity by enhancing the photosystem II machinery under MeJA application. Similar findings were observed by [55] where jasmonic acid application led to the accretion of plant pigments [56]. The results of the present study are also supported by different research groups who studied the impact of MeJA application, and the results showed that the treatment with MeJA led to increases in photosynthetic pigments under drought stress conditions compared to control plants [39,57,58,59,60]. When MeJA and AgNPs were used together, chlorophyll ab concentration reached a maximum after 72 h of exposure which was far more than the control. Hence, MeJA is proved to be an important determinant in enhancing chlorophyll and carotenoid contents in most plants [61,62].

Phenolic compounds play an important role in scavenging free radicals and protecting plants from the damaging effects of elevated ROS levels due to stress. A substantial upsurge in phenolic compounds by MeJA application has also been observed in various studies [63,64]. The maximum increase in phenolic compounds was observed in Giza 35 (soybean genotype) under MeJA treatment [1,65]. The authors of reference [66] also found that the formation of phenols in plant tissues increases under conditions of biotic stress [67]. Previous reports by the authors of reference [68] showed an increase in phenolic compounds under the effect of MeJA treatment. Therefore, our study suggested that MeJA could be used as a potential growth regulator to enhance the synthesis of these essential compounds in plants [68].

MeJA accumulates phenolic content in plant cells by regulating phenylpropanoid metabolism. MeJA stimulates three important enzymes in phenylpropanoid metabolism: Phenylalanine ammonia-lyase, cinnamate 4-hydroxylase, and 4-coumarin coenzyme A ligase [20]. Phenylalanine ammonia-lyase changes L-phenylalanine into trans-cinnamic acid, and then, trans-cinnamic acid is converted into coumaric acid by cinnamate 4-hydroxylase in the presence of oxygen molecules and NADPH. Later, coumaric acid is converted into 4-country-CoA by the enzyme 4-coumarin co-enzyme A ligase. Then, 4-coumaryl co-enzyme A further acts as a substrate for the synthesis of phenols. Moreover, the enzyme polyphenol oxidase is inhibited by MeJA. This enzyme oxidizes phenolic content into highly reactive quinones. With its inhibition, the phenolic content starts to accumulate and increase in concentration. In a study by the authors of reference [69], a considerable increase in antioxidant phenolic compounds and carotenoids was found while studying the effect of exogenous MeJA application on sweet potatoes. Another study performed on Romaine lettuce also showed similar results of increased phenolic content under the effect of MeJA [70]. In Braeburn apple, the phenolic content with some other bioactive compounds increased in concentration upon the exogenous application of MeJA [39].

Metallic nanoparticles have an impact on plant photosynthetic system structure and functions. Different plant species have shown a favorable impact of NPs on various photosynthetic metrics [58,59]. In *Linum usitatissimum* L., AgNPs increased photosynthetic pigments. Likewise, our findings of using AgNPs as elicitors are supported by the authors of reference [71] who studied the stimulatory effect of AgNPs on potted oriental lilies where they found the accumulation of chlorophyll a and b and carotenoids in the leaves of treated plants. Our results, which show a substantial increase in all photosynthetic pigments (chlorophyll a, chlorophyll b, carotenoids) in response to treatment with AgNPs, are in agreement with the results of the authors of reference [72].

According to the authors of reference [73], AgNPs considerably boost the photosynthesis process as a result of the altered nitrogen metabolism. Furthermore, the authors of reference [74] described that the chlorophyll content of corn plants was enhanced at low concentrations (10–50 μL/L) of AgNP treatment, whereas it was suppressed at higher concentrations of AgNPs. The authors of reference [67] reported that metallic nanoparticles induce chemical energy in photosynthetic systems. The higher contents of photosynthetic pigments, i.e., chlorophyll a, chlorophyll b, and carotenoids increase the rate of photosynthesis which, in turn, increases the weight and growth of the plant as observed in the study [67]. The author of reference [75] studied the effect of AgNPs on the fenugreek plant and discovered a rise in photosynthetic pigments as well as phenolics. The effect of biogenically generated AgNPs at appropriate concentrations on rice plant carotenoid and chlorophyll levels was explored by the authors of reference [76], and there was a considerable increase in their production. The authors of reference [77] investigated the effect of AgNPs on *Linum usitatissimum* L. and found that they were effective in raising carotenoid contents, and AgNPs showed a significant rise in chlorophyll concentration in recent research by the author of reference [78].

In the current study, *Aspergillus niger* was shown to be an effective elicitor for increasing photosynthetic pigments and total phenolics. According to a study on tomato plants, fungal elicitors showed an increase in chlorophyll, flavonoid, and total phenolic contents as well as plant dry mass [79]. A substantial rise in the chlorophyll content of plants treated with fungal elicitors was also observed. Another study by the authors of references [80,81,82,83] presented likewise results where the total phenolics were improved by *Aspergillus niger*. The authors of references [84,85,86,87] investigated the impact of *Aspergillus niger* on apricot (*Prunus armeniaca* L.) production and discovered a 30% increase in total phenolics. Similarly, the authors of reference [88] also found a significant increase in phenolics production in sunflower and soybean crops by the application of biotic elicitors. However, there has not been much research in this area in recent years, and this research might be of potential reference to help in this case.

Our results showed that the exogenous application of MeJA in hydroponics increased flavonoid contents, which is consistent with the findings of [89], which observed increased levels of quercetin and rutin in all soybean genotypes with MeJA application. Flavonoids play a role in plant defense mechanisms [90]. These results suggest that total flavonoid contents can be controlled and modified by MeJA application, which may help plants withstand different abiotic stresses [91]. MeJA has also been found to regulate secondary metabolism by stimulating the accumulation of flavonoids, alkaloids, and phenols in plant cells [92]. The methyl ester and jasmonic acid components of MeJA are believed to be involved in the synthesis of secondary metabolites like flavonoids [93]. Increased flavonoid and antioxidant activity were also observed in a study of blackberries by [94]. MeJA can regulate the expression of flavonoid genes in response to wounds, leading to increased flavonoid concentration [95]. MeJA is involved in the formation of 4-coumaryl coenzyme-A, which, when combined with malonyl coenzyme-A, forms naringeninchalcone in the presence of the enzyme naringeninchalcone synthase. Chalconoids, which are the precursors to flavonoids, have a structure similar to flavonoids, with two phenyl rings and a three-ringed structure. In a study on tomato plants, fungal elicitors increased chlorophyll contents, flavonoids, and total phenolic contents, as well as plant dry mass [96].

Lignin is a major component of plant cell walls and is a natural high molecular weight phenolic polymer with a complex composition and structure. It plays a significant role in plant growth, tissue and organ development, lodging resistance, and responses to various biotic and abiotic stresses. It also helps to prevent the spread of pathogens [97]. The elicitation of plants with elicitor molecules activates a series of defense responses, including the reinforcement of the cell wall through lignin deposition, the induction of defense enzyme activity, and the production of phenolics [98,99]. MeJA has been found to increase lignin accumulation in tea plants [100]. Histochemical staining has demonstrated the ability of AgNPs to induce lignin deposition in vascular bundles in *Triticum aestivum* L. Treatment with silver nanoparticles has also been shown to stimulate more intensive lignin accumulation in cell walls and improve the quality of in vitro-propagated *Thymus daenensis* Celak seedlings [101,102].

## 4. Materials and Methods

### 4.1. Plant Material, Experimental Design, Sterilization of Seeds and Sand

Seeds of *S. marianum* were obtained from the National Agriculture Research Centre (NARC) in Islamabad, Pakistan. The seeds were disinfected with 70% ethanol (C_2_H_5_OH) for 1 min after being washed with distilled water initially. Washing with alcohol was performed, and to remove any remaining ethanol, the seeds were once again washed 3 times with distilled water. Next, 0.01% mercuric chloride solution was employed for two min, and 4 to 5 repetitions of distilled water washing were then performed (81–82). The sand used in the study was also sterilized. For this purpose, it was repeatedly washed with distilled water. After rinsing, it was addressed with sodium hypochlorite (NaOCl) for 5 min and then washed to completely remove any sodium hypochlorite traces, repeating the process three to five times with distilled water. The sand was then dried in an oven set to 70 °C [103].

### 4.2. Seed Germination and Shifting to Hydroponic System

After sowing the seeds in sterile sand, the pots were kept under controlled conditions (24–25 °C, 20–700 µMol m^−2^s^−1^). The seedlings received regular watering and Hoagland solution supplements after emergence. After a fortnight of germination, the plants were reduced to 1 plant per pot and placed in a hydroponic system [104]. A setup of an air pump was added to ensure a continuous supply of oxygen [105]. The seedlings were then given 7 distinct treatments to develop in, 1 of which was designated as control (Table 1). Different elicitors were used for each treatment, whereas the control solution, devoid of the elicitors, used solely Hoagland solution as a supplement. In a completely randomized design (CRD), 5 replications for every treatment were used. For each treatment, thirty plants were placed in a hydroponic system for this objective. For each treatment, six pickups were performed, and 5 seedlings were cultivated for each harvest. The 1st harvest was completed after one day of treatment; the 2nd was completed after two days; the 3rd was completed after 3 days; the 4th was completed after 96 h; the 5th was completed after 120 h; and the 6th harvest was completed after 144 h of therapy. The treatment procedure started on 19 May 2022 (12:00 pm) and ended on 25 May 2022 (12:00 pm). The fresh weight of the leaves was measured in grams using an electronic balance.

**Figure 6:**
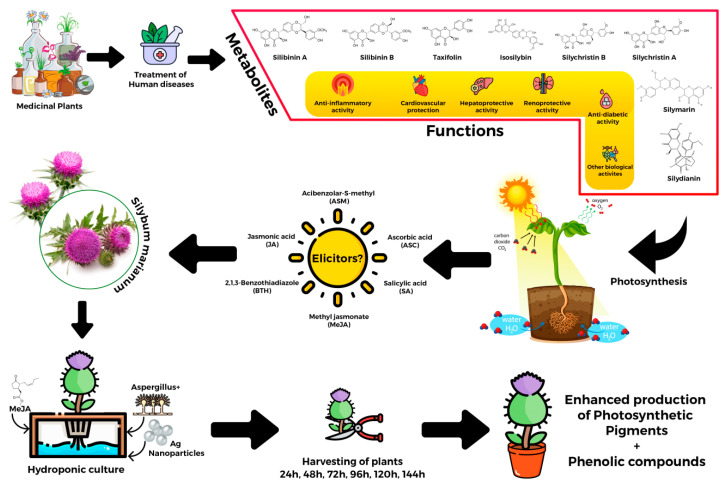
Schematic representation of experiments conducted for study in hydroponics.

In the research, various elicitors were applied to encourage the synthesis of secondary products. To do this in the hydroponic medium, the addition of methyl jasmonate (Sigma-Aldrich, Waltham, MA, USA) (100 µM/L), fungal elicitors (200 mg/L), and sonicated green-synthesized AgNPs (1 ppm/L) was performed singly and as a mixture of all these compounds. The green synthesis of AgNPs was performed by the reaction of silver nitrate and *Fortunella margarita* by slight modification in the method as described by the authors of reference [106]. For every hydroponic replication, Hoagland’s solution was already added to the pots (Figure 6). Different treatments of elicitors and their combinations are shown in Table 1. A controlled environment was provided to the plants with 24–25 °C of temperature. A pictorial representation of the study in question has been presented in Figure 1.

### 4.3. Phytochemical Analyses

The plants were exposed to phytochemical evaluations after harvest. The plant leaves were examined for a variety of phytochemical assays, including those for phenolic, carotenoid, and chlorophyll contents.

### 4.4. Chlorophyll and Carotenoid Content

Chlorophyll (a, b) and carotenoid contents were determined according to the authors of reference [107]. One gram of finely chopped fresh leaves was ground with 20–40 mL of acetone at a concentration of 80%. The sample was centrifuged for 5 min at 5000–10,000 rpm. The supernatant was transferred, and the operation was repeated until the residue was colorless, at which point, the absorbance was measured in a spectrophotometer at 663 and 645 nm. Chlorophyll a and Chlorophyll b concentrations were calculated using the formulae below for total chlorophyll and carotenoids.

$$\mathrm{Chlorophyll}\;a\;\left(µg/g\;\mathrm{FW}\right)=\{12.7\;(\mathrm{OD}663-2.69\;\left(\mathrm{OD}645\right)\times \;V/1000\times \;W\}$$

$$\mathrm{Chlorophyll}\;b\;\left(µg/g\;\mathrm{FW}\right)=\{12.9\;(\mathrm{OD}645-4.68\;\left(\mathrm{OD}663\right)\times \;V/1000\times \;W\}\}$$

$$\mathrm{Total}\;\mathrm{chlorophyll}\;\left(µg/g\;\mathrm{FW}\right)=[20.2\;\left(\mathrm{OD}645-8.02\;\left(\mathrm{OD}663\right)\times \;V/1000\times \;W\right]$$

$$\mathrm{Carotenoids}\;\left(µg/g\;\mathrm{FW}\right)=\;\mathrm{OD}480+\left(0.114\times \;\mathrm{OD}663\right)-\left(0.638\times \;\mathrm{OD}645\right)$$

where, OD = Optical density; V = Volume of sample; W = Fresh weight of sample

### 4.5. Total Phenolic Content

The Folin–Ciocalteu method was slightly modified to determine the entire phenol concentration [108]. One gram of crushed leaves was homogenized in 80 percent aqueous ethanol at room temperature, and centrifuged in cold at 10,000× *g* for 15 min. The supernatants were collected, deposited on evaporating plates, and dried at room temperature after two extractions with 80 percent ethanol. The residue was 5 mL of Folin–Ciocalteu reagent (previously diluted to 1:10 *v*/*v* with water) and 4 mL (75 g/L) of sodium carbonate (Na_2_CO_3_). Vortexing the material for 15 s was performed, and it was incubated at 40 °C for thirty minutes for color development. The absorbance was measured at 765 nanometers by a spectrophotometer, and the amount of total phenol content was calculated in mg/g of tannic acid (C_76_H_52_O_46_) equivalent using the following equation based on the calibration curve:

*y* = 0.1216*x*, *r*2 = 0.9365,

where *x* is the absorbance and *y* is the tannic acid equivalent (mg/g).

### 4.6. Determination of Lignin Content

Lignin has antioxidant and antimicrobial properties and has potential use in the production of pharmaceuticals and biomedical applications. Its biocompatibility, ability to absorb UV light, antioxidant and antimicrobial activities, and ability to improve the mechanical strength of processed biomaterials make it a promising candidate for pharmaceutical and biomedical applications. Research suggests that lignin could be used in the development of biomedical hydrogels, drug-delivery vehicles, or bio composites for tissue engineering and wound healing [26].

### 4.7. Determination of Flavonoids

To measure the total flavonoid content, a protocol developed by the authors of reference [24] was followed. The extract was placed in a 10 mL volumetric flask, and distilled water was added to forma volume of 5 mL. After 5 min, 0.3 mL of sodium nitrite (NaNO_2_) and 0.3 mL of aluminum chloride (AlCl_3_) were added. After an additional 6 min, 2 mL of 1 M sodium hydroxide (NaOH) was added, and the volume was brought up to 10 mL with distilled water. The solution was mixed well, and the absorbance was measured using a spectrophotometer at 510 nm, with a blank as the reference. A calibration curve was used to determine the flavonoid content, which was expressed as a percentage of quercetin (C_15_H_10_O_7_) in the extract [25].

### 4.8. Experimental Design and Statistical Analysis

The experiment was conducted in five replicates for each treatment, including a control, using a completely randomized design (CRD). The plants were harvested at 24 h intervals up to 144 h of exposure. Data calculations were completed using Microsoft Excel 2019^®^, and Origin 2021 b was used for one-way ANOVA statistical analysis. The Tukey test at the 0.05 probability level was used to determine whether there was a difference between the treatment means. Pearson correlation among treatments and variables and principal component analysis were also performed using Origin 2021b [109].

## 5. Conclusions

The results of this study show that several elicitors, such as the fungus *Aspergillus niger* (0.2 g/L), methyl jasmonate (100 M/L), and silver nanoparticles (1 ppm), had a favorable influence on the levels of photosynthetic pigments and total phenolics, both alone and in combination. Photosynthetic pigments were produced in significant amounts under MeJA, AgNPs, and a combination of MeJA and fungal elicitors and MeJA and AgNPs. Carotenoid production was also substantial under MeJA and a combination of MeJA and AgNPs and MeJA and fungal elicitors, respectively. However, in the case of total phenolics, AgNPs, a combination of meJA and fungal elicitors, and MeJA treatment showed the best result among all other combinations. We may, therefore, infer that using elicitors to treat *S. marianum* in hydroponic culture could be a useful method for researching how plants quickly increase metabolite synthesis in reaction to diverse stress events to control their metabolism. Increasing the production of medicinally important bioactive molecules and compounds is the need of the hour. It is, therefore, recommended that the elicitors which produced promising results can be used for further studies in different concentrations and on different pharmacologically important plants to augment the synthesis of phytocompounds that have medicinal and therapeutic importance. In contrast to other biotechnological processes, hydroponics is inexpensive and less time-consuming. These elicitors ought to be used with a variety of medicinally effective crops cultivated in a variety of growth mediums and environments, such as tissue culture, sand culture, Petriplate culture, soil culture, and hydroponics. Additionally, there is avariety of other kinds of nanoparticles in a range of sizes that can be used in numerous investigations. Hence, extraordinary results might be achieved in future studies with the use of various cultural techniques and types of elicitors. Furthermore, a study on the combined application of elicitors is required, as novel compositions of potential elicitors might be explored by experimenting with different combinations of elicitors.

## Figures and Tables

**Table 1:** All the treatments with tags used for the experiment.

Sr. No.	Treatments	Tags
1	Control	T0
2	MeJA	T1
3	Fungal elicitors	T2
4	Green-synthesized AgNPs	T3
5	MeJA + Fungal elicitors	T4
6	MeJA + Green-synthesized AgNPs	T5
7	Fungal elicitors + Green-synthesized AgNPs	T6
8	Fungal elicitors + Green-synthesized AgNPs + MeJA	T7

## Data Availability

Not applicable.
